# High Fat Diets Sex-Specifically Affect the Renal Transcriptome and Program Obesity, Kidney Injury, and Hypertension in the Offspring

**DOI:** 10.3390/nu9040357

**Published:** 2017-04-03

**Authors:** You-Lin Tain, Yu-Ju Lin, Jiunn-Ming Sheen, Hong-Ren Yu, Mao-Meng Tiao, Chih-Cheng Chen, Ching-Chou Tsai, Li-Tung Huang, Chien-Ning Hsu

**Affiliations:** 1 Department of Pediatrics, Kaohsiung Chang Gung Memorial Hospital and Chang Gung University College of Medicine, Kaohsiung 833, Taiwan; tainyl@hotmail.com (Y.-L.T.); ray.sheen@gmail.com (J.-M.S.); yuu2004taiwan@yahoo.com.tw (H.-R.Y.); tmm@cgmh.org.tw (M.-M.T.); charllysc@cgmh.org.tw (C.-C.C.); litung.huang@gmail.com (L.-T.H.); 2 Institute for Translational Research in Biomedicine, Kaohsiung Chang Gung Memorial Hospital and Chang Gung University College of Medicine, Kaohsiung 833, Taiwan; 3 Department of Obstetrics and Gynecology, Kaohsiung Chang Gung Memorial Hospital and Chang Gung University College of Medicine, Kaohsiung 833, Taiwan; lyu015ster@gmail.com (Y.-J.L.); nick@cgmh.org.tw (C.-C.T.); 4 Department of Traditional Chinese Medicine, Chang Gung University, Linkow 244, Taiwan; 5 Department of Pharmacy, Kaohsiung Chang Gung Memorial Hospital, Kaohsiung 833, Taiwan; 6 School of Pharmacy, Kaohsiung Medical University, Kaohsiung 807, Taiwan

**Keywords:** clock gene, developmental origins of health and disease (DOHaD), high-fat diet, hypertension, next generation sequencing, nitric oxide, kidney disease, oxidative stress, renin-angiotensin system

## Abstract

Obesity and related disorders have increased concurrently with an increased consumption of saturated fatty acids. We examined whether post-weaning high fat (HF) diet would exacerbate offspring vulnerability to maternal HF-induced programmed hypertension and kidney disease sex-specifically, with a focus on the kidney. Next, we aimed to elucidate the gene–diet interactions that contribute to maternal HF-induced renal programming using the next generation RNA sequencing (NGS) technology. Female Sprague-Dawley rats received either a normal diet (ND) or HF diet (D12331, Research Diets) for five weeks before the delivery. The offspring of both sexes were put on either the ND or HF diet from weaning to six months of age, resulting in four groups of each sex (maternal diet/post-weaning diet; *n* = 5–7/group): ND/ND, ND/HF, HF/ND, and HF/HF. Post-weaning HF diet increased bodyweights of both ND/HF and HF/HF animals from three to six months only in males. Post-weaning HF diet increased systolic blood pressure in male and female offspring, irrespective of whether they were exposed to maternal HF or not. Male HF/HF offspring showed greater degrees of glomerular and tubular injury compared to the ND/ND group. Our NGS data showed that maternal HF diet significantly altered renal transcriptome with female offspring being more HF-sensitive. HF diet induced hypertension and renal injury are associated with oxidative stress, activation of renin-angiotensin system, and dysregulated sodium transporters and circadian clock. Post-weaning HF diet sex-specifically exacerbates the development of obesity, kidney injury, but not hypertension programmed by maternal HF intake. Better understanding of the sex-dependent mechanisms that underlie HF-induced renal programming will help develop a novel personalized dietary intervention to prevent obesity and related disorders.

## 1. Introduction

The growing prevalence of obesity has a profound impact on worldwide health, including risk of hypertension and kidney disease. Obesity may originate from the early life. Pre- and post-natal nutrition together influence developmental programming, leading to disease in adulthood [1]. Obesity and related disorders have increased concurrently with an increased consumption of saturated fatty acids [2]. High-fat (HF) diets have been generally used to generate animal models for obesity and related disorders [3,4,5]. In this regard, maternal HF intake leads to a variety of chronic diseases in adult offspring, including obesity, hypertension, and kidney disease [3,4,6,7].

The kidney controls blood pressure (BP) and plays a crucial role in the development of hypertension [8], thus renal programming is considered a key mechanism for programmed hypertension and kidney disease [8,9,10,11]. A number of mechanisms have been proposed to interpret renal programming, including oxidative stress, inappropriate activation of the renin-angiotensin system (RAS), and impaired tubular sodium handling [8,9,10,11]. Additionally, renal circadian clocks are involved in the sodium balance and BP control [12]. Disturbances of circadian clocks increase the risk of a variety of metabolic diseases [13]. Despite a previous study showing that HF diet causes dysregulated circadian clock in the liver and kidney [14], exactly how the circadian clock is programmed by maternal and post-weaning HF intake is unclear. Given that HF diet has been reported to mediate oxidative stress, RAS, sodium transporters, and circadian clock [5,14,15,16], we hypothesized that post-weaning HF intake enhances offspring vulnerability to maternal HF-induced programmed hypertension and kidney disease via mediating these mechanisms described above.

Sex differences have been observed in obesity, hypertension, and kidney disease [17,18,19]. However, it is unclear whether sex differences exist in maternal HF plus post-weaning HF consumption induced hypertension and kidney injury. Previously, our study showed that prenatal dexamethasone induced programmed hypertension and alterations of renal transcriptome in a sex-specific manner [20]. Additionally, we demonstrated that post-weaning HF diet exacerbated hypertension programmed by early dexamethasone exposure in adult male offspring [21]. However, to what extent maternal HF diet adversely affects the kidney to post-weaning HF intake in adult offspring and whether there exists sex-specific susceptibility is unclear. Although nutrigenetics and nutrigenomics have been introduced to understand existing interactions between genes and diets [22,23], very limited studies have analyzed the transcriptome of the offspring kidneys in response to maternal diets and examined their relationships to programmed hypertension and kidney disease [24,25]. We, hence, further employed the whole-genome RNA next-generation sequencing (NGS) to quantify the abundance of RNA transcripts in the one-week-old offspring kidney from maternal exposure to HF diet.

## 2. Materials and Methods

### 2.1. Experimental Design

This study was carried out in strict accordance with the recommendations of the Guide for the Care and Use of Laboratory Animals of the National Institutes of Health. The protocol was approved by the Institutional Animal Care and Use Committee of the Kaohsiung Chang Gung Memorial Hospital. Virgin Sprague-Dawley (SD) rats (BioLASCO Taiwan Co., Ltd., Taipei, Taiwan) were housed and maintained in a facility accredited by the Association for Assessment and Accreditation of Laboratory Animal Care International. International. The rats were exposed to a 12 h light/12 h dark cycle. Male SD rats were caged with female rats until mating was confirmed by examining vaginal plug.

Female rats were weight-matched and assigned to receive either a normal diet with regular rat chow (ND; Fwusow Taiwan Co., Ltd., Taichung, Taiwan; 52% carbohydrates, 23.5% protein, 4.5% fat, 10% ash, and 8% fiber) or high-fat hypercaloric diet (HF; D12331, Research Diets, Inc., New Brunswick, NJ, USA; 58% fat (hydrogenated coconut oil) plus high sucrose (25% carbohydrate)) ad libitum for 5 weeks before mating and during gestation and lactation. After birth, litters were culled to give equal numbers of males and females for a total of eight pups to standardize the received quantity of milk and maternal pup care. Three male and three female offspring from each group (control and HF) were killed at 1 week of age. Their kidneys were isolated for NGS analysis. The remaining offspring were assigned to four experimental groups of each sex (maternal diet/post-weaning diet; *n* = 5–7/group): ND/ND, ND/HF, HF/ND, and HF/HF. The offspring of both sexes were weaned at 3 weeks of age, and onto either the normal diet (ND) or HF diet ad libitum from weaning to 6 months of age. BP was measured in conscious rats at 4, 8, 12, 16, 20, and 24 weeks of age by using an indirect tail-cuff method (BP-2000, Visitech Systems, Inc., Apex, NC, USA) after systematically trained. To ensure accuracy and reproducibility, the rats were acclimated to restraint and tail-cuff inflation for 1 week before the experiment, and measurements were taken at 1:00 PM to 5:00 PM each day. Rats were placed on the specimen platform, and their tails were passed through tail cuffs and secured in place with tape. After a 10-minute warm up period, 10 preliminary cycles were performed to allow the rats to adjust to the inflating cuff. For each rat, 5 measurements were recorded at each time point as previously described [20]. Three stable consecutive measures were taken and averaged. 

At 6 months of age, offspring were sacrificed in the early light phase of the light–dark cycle. Rats were anesthetized by intraperitoneally injecting ketamine (50 mg/kg body weight) and xylazine (10 mg/kg body weight) and were euthanized by intraperitoneally injecting an overdose of pentobarbital. The midline of the abdomen was opened. The aorta was cannulated with a 20–23-gauge butterfly needle, blood samples were collected, the vena cava was cut, and PBS was perfused until the kidneys were blanched. Kidneys were harvested after perfusion, divided into cortex and medulla, and stored at −80 °C for further analysis.

### 2.2. Biochemical Analysis

The blood concentrations of total cholesterol, high-density lipoprotein (HDL), triglyceride, glucose, and aspartate transaminase (AST) and alanine aminotransferase (ALT) activities were determined by a standard autoanalyzer (Hitachi model 7450, Tokyo, Japan). Intraperitoneal glucose tolerance test (IPGTT) was performed as previously described [26]. After an 8-h fast, blood samples were collected at five time points: before injection and at 15, 30, 60, and 120 min after the intraperitoneal injection of glucose (2 g/kg body weight). Plasma glucose levels were immediately measured using the enzymatic (hexokinase) method with a glucose assay kit. Plasma NOx (NO^−^ + NO3^−^) levels were measured by the Griess reaction as previously described [27]. 

### 2.3. Histology and Morphometric Study

Histology was performed on 4 μm sections of formalin-fixed kidney, blocked in paraffin wax and stained with periodic acid-Schiff (PAS). The level of renal injury was assessed on a blinded basis by calculating glomerular and tubulointerstitial injuries that we described previously [27]. Up to one hundred glomeruli were scored based on the 0 to 4+ injury scale, to calculate the glomerular injury score. Tubulointerstitial injury (TI) scores were based on the presence of tubular cellularity, basement membrane thickening, dilation, atrophy, sloughing, or interstitial widening. TI scores were graded as follows: 0, no changes; grade 1, <10% TI involvement; grade 2, 10%–25% TI involvement; grade 3, 25%–50% TI involvement; grade 4, 50%–75% TI involvement; and grade 5, 75%–100% TI involvement.

### 2.4. Detection of l-arginine, l-citrulline, ADMA, and SDMA by HPLC

Plasma l-arginine, l-citrulline, and asymmetric dimethylarginine (ADMA, an endogenous inhibitor of nitric oxide synthase) levels were measured using high-performance liquid chromatography (HP series 1100; Agilent Technologies Inc., Santa Clara, CA, USA) with the o-phtalaldehyde-3-mercaptoprionic acid derivatization reagent described previously [28]. Standards contained concentrations of 1–100 mM l-arginine, 1–100 mM l-citrulline, 0.5–5 mM ADMA, and 0.5–5 mM SDMA. The recovery rate was approximately 95%.

### 2.5. Next-Generation Sequencing and Analysis

As we described previously [24], kidney cortex samples (*n* = 3/group) were pooled for whole-genome RNA NGS analysis and performed by Welgene Biotech Co., Ltd. (Taipei, Taiwan). All procedures were performed according to the Illumina protocol. For all samples, library construction was performed using the TruSeq RNA Sample Prep Kit v2 for ~160 bp (single-end) sequencing and the Solexa platform. Gene expression was quantified as fragment per kilobase of exon per million mapped fragment (FPKM). Cufflink v 2.1.1 and CummeRbund v 2.0.0 (Illumina Inc., San Diego, CA, USA) were used to perform statistical analyses of the gene expression profiles. Gene Ontology (GO) term enrichment and fold enrichment or depletion for gene lists of significantly up- and down regulated genes in kidney were determined. The reference genome and gene annotations were retrieved from Ensembl database. GO analysis for significant genes was performed using Kyoto Encyclopedia of Genes and Genomes (KEGG) and NIH DAVID Bioinformatics Resources 6.8 (NIH, Bethesda, MD, USA) to identify regulated biological themes [29].

### 2.6. Quantitative Real-time Polymerase Chain Reaction (PCR)

RNA was extracted using TRIzol reagent treated with DNase I (Ambion, Austin, TX, USA) to remove DNA contamination, and reverse transcribed with random primers (Invitrogen, Carlsbad, CA, USA) [28]. RNA concentration and quality were checked by measuring optical density at 260 and 280 nm. The complementary DNA (cDNA) product was synthesized using a MMLV Reverse Transcriptase (Invitrogen). Two-step quantitative real-time PCR was conducted using the QuantiTect SYBR Green PCR Kit (Qiagen, Valencia, CA, USA) and the iCycler iQ Multi-color Real-Time PCR Detection System (Bio-Rad, Hercules, CA, USA). First, the kidney fibrotic markers, extracellular matrix collagen I and α-smooth muscle actin (α-SMA) were analyzed. Next, components of RAS analyzed in this study included renin (*Ren*); (pro)renin receptor (*Atp6ap2*), angiotensinogen (*Agt*), angiotensin converting enzyme-1 and -2 (*Ace* and *Ace2*), angiotensin II type 1 and 2 receptor (*Agtr1a* and *Agtr2*), and angiotensin (1–7) receptor *Mas1*. Moreover, several core clock genes in the feedback loop were studied, including *Clock* and *Bmal1* of the positive limb; and *Cry1, Cry2, Per1, Per2*, and *Per3* of the negative limb. In addition to these, other clock genes or clock-controlled genes, such as *Ck1e* and *Nr1d1* were analyzed. The 18S rRNA gene (*Rn18s*) was used as a reference. Sequences of primers used in this study are provided in Table 1. All samples were run in duplicate. To quantify the relative gene expression, the comparative threshold cycle (CT) method was employed. For each sample, the average CT value was subtracted from the corresponding average r18S value, calculating the ΔCT. ΔΔCT was calculated by subtracting the average control ΔCT value from the average experimental ΔCT. The fold-increase of the experimental sample relative to the control was calculated using the formula 2^−ΔΔCT^.

### 2.7. Western Blot

Western blot analysis was performed as previously described [28]. Sodium hydrogen exchanger type 3 (NHE3), Na^+^/Cl^−^ cotransporter (NCC), Na-K-2Cl cotransporter (NKCC2), and Na^+^/K^+^-ATPase a 1 subunit (NaKATPase) were analyzed by incubating the samples overnight with the following antibodies: rabbit anti-rat antibody for NHE3 (1:1000 dilution; Alpha Diagnostic Intl Inc., San Antonio, TX, USA), rabbit anti-rat antibody for NCC (1:2000 dilution; Merck Millipore, Billerica, MA, USA), rabbit anti-rat antibody for NKCC2 (1:1000 dilution; Alpha Diagnostic Intl Inc.), and mouse antibody for NaKATPase (1:10,000 dilution; Abcam, Cambridge, MA, USA). Bands of interest were visualized using ECL reagents (PerkinElmer, Waltham, MA, USA) and quantified by densitometry (Quantity One Analysis software; Bio-Rad), as integrated optical density (IOD) after subtraction of background. The IOD was factored for Ponceau red staining to correct for any variations in total protein loading. The protein abundance was represented as IOD/PonS.

### 2.8. Immunohistochemistry Staining for 8-OHdG

8-Hydroxydeoxyguanosine (8-OHdG) is a DNA oxidation product that was measured to assess DNA damage. Paraffin-embedded tissue sectioned at a thickness of 2 μm was deparaffinized in xylene and rehydrated in a graded ethanol series to phosphate-buffered saline. Immunohistochemical staining was performed using anti-8-OHdG antibody (1:2500; Santa Cruz Biotechnology, Dallas, TX, USA) with a SuperSensitive polymer-horseradish peroxidase immunohistochemistry detection kit (BioGenex, San Ramon, CA, USA) as we described previously [27]. Identical staining without the primary antibody was used as a negative control.

### 2.9. Statistical Analysis 

All data are expressed as mean ± SEM. Parameters were compared using two-way analysis of variance (ANOVA) followed by a Tukey’s post hoc test for multiple comparisons. Weights, metabolic and plasma parameters among the groups were further analyzed by one-way ANOVA with a Tukey’s post hoc test. A *P*-value < 0.05 was considered statistically significant. All analyses were performed using the Statistical Package for the Social Sciences software (SPSS Inc., Chicago, IL, USA).

## 3. Results

### 3.1. Morphological Features and Biochemistry

**Figure 1:**
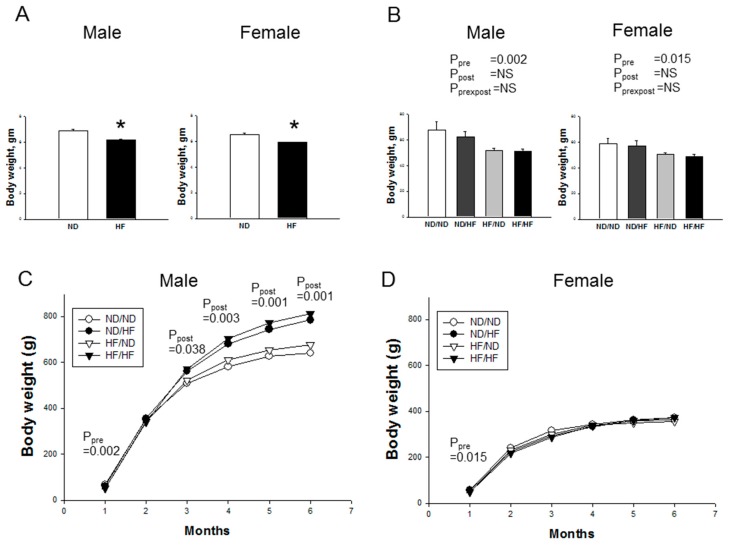
Effects of maternal and postnatal high-fat (HF) diet on bodyweight in: (**A**) neonates; (**B**) male and female offspring at one month of age; and (**C**) male offspring; and (**D**) female offspring from one to six months. * *p* < 0.05 vs. HF; Pre × Post, interaction of pre × post; NS, not significant; *N* (pups/L) = 5-7/3 per group.

There were no differences in the litter size (ND = 14 ± 0.8; HF = 15.5 ± 0.8) and ratio of male-to-female pups (ND vs. HF = 0.84 vs. 1.14). One male pup died at Postnatal Day 5 in the ND/ND group, while the mortality rate was 0% in the other groups. The birth body weight was lower in HF offspring compared to ND offspring in both sexes (Figure 1A). HF offspring born with intrauterine growth restriction (IUGR) continued to have lower body weight until one month of age in both sexes (Figure 1B). In males, post-weaning HF diet increased BW of both ND/HF and HF/HF animals from three to six months (Figure 1C). In contrast, significant BW gain was not shown in female offspring fed with HF diet (Figure 1D). 

At six months of age, either maternal or post-weaning HF has no effect on kidney weight of each sex. There was a significant effect of post-weaning HF diet on the kidney weight-to-body weight ratio in males (*P*_post_ < 0.01). As compared to ND/ND group, both maternal and post-weaning HF intake increased plasma levels of AST and ALT in both sexes (Table 2). Male offspring exposed to post-weaning HF consumption showed highest plasma levels of total cholesterol among the four groups. There was little measurable effect of either maternal or post-weaning HF diet on plasma levels of triglyceride, HDL, and glucose in offspring of both sexes. In female offspring, plasma triglyceride levels were higher in HF/ND group than those in ND/HF group. The increase in the glucose area under curve (AUC) after an IPGTT was found in ND/HF group compared to controls in females.

### 3.2. Blood Pressure and Renal Outcome

**Figure 2:**
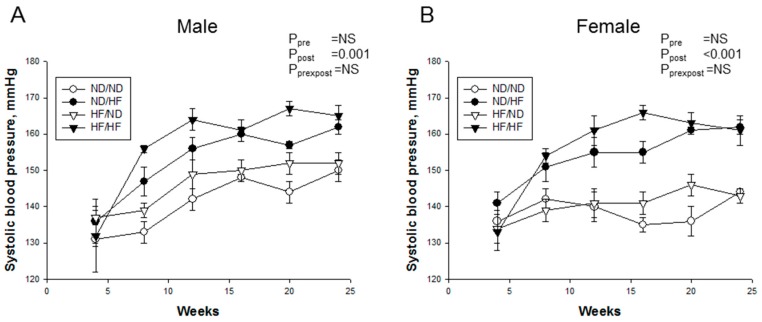
Effects of maternal and postnatal high-fat (HF) diet on systolic blood pressure in: (**A**) male; and (**B**) female offspring from four to 24 weeks. Pre × Post, interaction of pre × post; NS, not significant; *N* (pups/L) = 5-7/3 per group.

Longitudinal measurement of systolic BP from four to 24 weeks of age showed that post-weaning HF diet increased SBP in male (Figure 2A, *P*_post_ = 0.001) and female offspring (Figure 2B, *P*_post_ < 0.001), irrespective of whether they were offspring of dams with maternal HF or not. 

**Figure 3:**
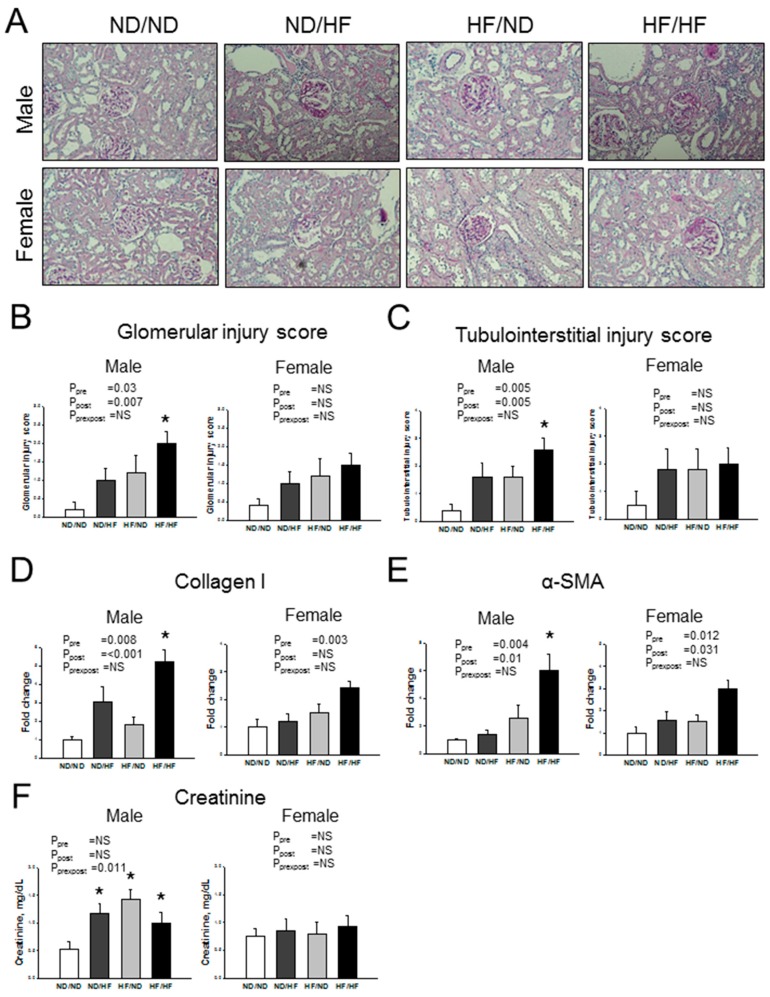
Maternal and post-weaning HF diet induced greater degrees of kidney injury in male than female offspring. Effects of maternal and post-weaning HF diet on: (**A**) morphological changes; (**B**) glomerular injury; (**C**) tubulointerstitial injury; (**D**) mRNA expression of collagen I; (**E**) α-smooth muscle actin (α-SMA); and (**F**) creatinine level. Pre × Post, interaction of pre × post; NS, not significant; * *p* < 0.05 vs. ND/ND; *N* (pups/L) = 5-7/3 per group.

Consistent with previous reports [30,31], male offspring exposed to post-weaning HF showed glomerulosclerosis, segmental necrosis, thickening in the basal membrane of glomeruli and tubules, dilatation in glomerular capillaries, and tubular dilatation (Figure 3A). Maternal and post-weaning HF were associated with greater degrees of glomerular (Figure 3B, *P*_pre_ = 0.03 and *P*_post_ = 0.007) and tubulointerstitial injury (Figure 3C, *P*_pre_ = 0.005 and *P*_post_ = 0.005) in male offspring kidneys than those in females. Consistent with the histologic findings, compared with the ND/ND group, HF/HF group exhibited significantly increased extracellular matrix mRNA expression of collagen I and α-smooth muscle actin (α-SMA) (Figure 3D,E) in males. Additionally, maternal and post-weaning HF synergistically caused a higher creatinine level in HF/HF group compared with ND/ND group in males. (Figure 3F, *P*_prexpost_ = 0.011). However, plasma creatinine level was not different among the four groups in females. These data demonstrated that maternal and post-weaning HF-induced kidney injury mainly in male but not female offspring at 24 weeks of age.

### 3.3. Renal Transcriptome

We next analyzed differential gene expression induced by maternal HF consumption in the kidney. Among the differential expressed genes (DEGs), a total of 21 genes (five up- and 16 downregulated genes by male HF versus male ND, Table S1) met the selection criteria of: (i) genes that changed by FPKM > 0.3; and (ii) minimum of twofold difference in normalized read counts between group. As shown in Table S2, a total of 251 DEGs (154 up- and 97 downregulated genes by female HF versus female ND) were noted in response to maternal HF exposure in female offspring. Among them, a total of nine shared genes were identified: *Afp, Cubn, Dgkg, Kcnj15, Lrp2, Slc4a4, Slc6a19, Slc15a1,* and *Stra6*. The DEGs between males and females were further analyzed. There were 91 (67 male-biased genes vs. 24 female-biased genes) and nine (two male-biased genes vs. seven female-biased genes) genes by male versus female that reached a minimum of twofold difference between sexes in the ND group (Table S3) and HF group (Table S4), respectively. 

We next used DAVID v6.8 [29] to find functionally related gene groups and gain biological insight from our gene lists. We found one and five signaling pathways identified as the significant KEGG pathways in the male and female offspring kidneys exposed to maternal HF, respectively (Table 3). These KEGG pathways include oxidative phosphorylation, protein digestion and absorption, metabolic pathways, ribosome, and cardiac muscle contraction. Even though none of these genes were related to regulation of BP by GO analysis, we observed four genes with at least twofold difference between HF vs. control in female: *Agtr1b* (fold change (FC) = 4.4) and *Ace* (FC = 0.3) in the RAS, *Ddah1* (FC = 0.3) in the NO system, and *Slc12a3* (FC = 0.3) belonging to sodium transporters.

Because oxidative stress, RAS pathway, and sodium transporters are involved in renal programming [8,9,10,11], and because our NGS data demonstrated that some components of these pathways were altered in response to maternal HF intake, we further investigated these pathways to elucidate underlying mechanisms related to programmed hypertension and kidney disease.

### 3.4. Oxidative Stress and Nitric Oxide Pathway

**Figure 4:**
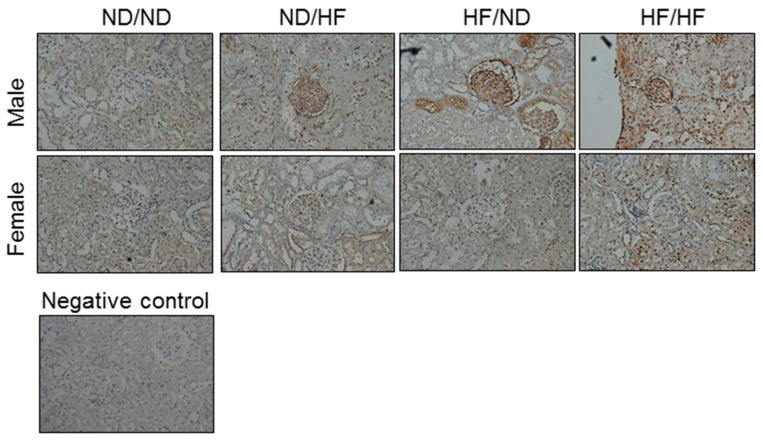
Light micrographs illustrating immunostaining for 8-OHdG in the offspring kidney (400×).

We evaluated oxidative stress in the kidney by immunostaining 8-OHdG, an oxidative DNA damage marker. As shown in Figure 4, immunostaining of both cytoplasmic and nuclear 8-OHdG in the glomeruli and renal tubules indicated little staining in the ND/ND group, an intermediate level of staining in the ND/HF as well as HF/ND groups, and intense staining in the HF/HF group in males. Unlike males, females showed little 8-OHdG staining in the ND/HF, HF/ND, and HF/HF groups. 

The link between oxidative stress and NO deficiency in programmed hypertension and kidney disease has been recognized [10,11]. We, hence, further investigated whether HF diet induced an imbalance in the NO pathway (Table 4). Plasma level of l-citrulline, a precursor of l-arginine, was higher in post-weaning HF treated groups in both sexes. Either maternal or post-weaning HF diet decreased plasma l-arginine level in males (*P*_pre_=0.041 and *P*_post_ < 0.001), while only post-weaning HF had an effect to reduce plasma l-arginine level in females (*P*_post_ = 0.027). Although maternal HF increased plasma ADMA level in males (*P*_pre_ = 0.001), there was a significant effect of post-weaning HF with decreased plasma ADMA level in males (*P*_post_ = 0.003) and females (P_post_ = 0.001). Maternal HF induced a higher plasma SDMA, an indirect inhibitor of nitric oxide synthase, level in HF/ND group compared to ND/ND in males. Post-weaning HF caused a reduction of plasma SDMA level in females. In male offspring, post-weaning HF diet induced a lower l-arginine-to-ADMA ratio (*P*_post_ < 0.001), a marker representing NO bioavailability, which was accompanied by an interaction between maternal and post-weaning HF (*P*_prexpost_ = 0.011). Similarly, post-weaning HF decreased plasma NOx level in males (*P*_post_ = 0.001). Taken together, our findings suggest sex-dependent renal programming associated with a greater degree of oxidative stress and a lower NO bioavailability in male than in female offspring kidney in response to HF consumption.

### 3.5. RAS and Sodium Transporters

**Figure 5:**
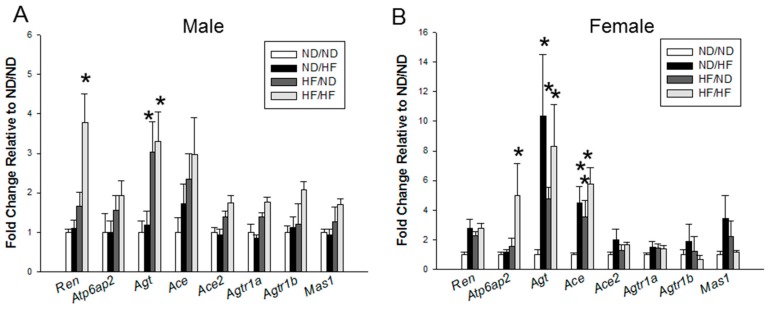
Effects of maternal and postnatal high-fat diet (HF) on gene expression of RAS components in: (**A**) male; and (**B**) female offspring. * *p* < 0.05 vs. ND/ND.

We evaluated the renal mRNA expression of RAS components (Figure 5). Renal mRNA expression of *Ren* was higher in the HF/HF group than in ND/ND group. In females, HF/HF group had higher mRNA expression of *Atp6ap2* in the kidney compared to ND/ND group. Maternal HF significantly increased *Agt* expression in both sexes. In females, both maternal and post-weaning HF significantly increased the renal mRNA expression of *Ace* in the ND/HF, HF/ND, and HF/HF groups compared with those in ND/ND group (Figure 5B). However, downstream signals of the RAS, such as *Agtr1a, Agtr1b*, and *Mas1*, were not different among the four groups of both sexes. 

**Figure 6:**
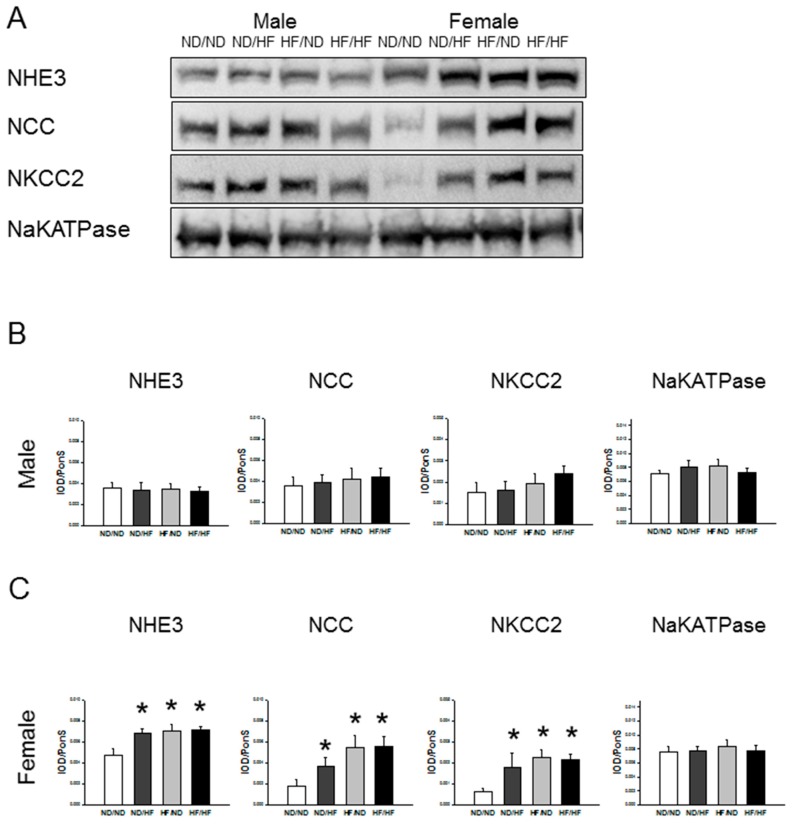
Effects of maternal and postnatal high-fat diet (HF) on sodium transporters expression in male and female offspring. (**A**) Representative Western blots of NHE3 (90 kDa), NCC (130 kDa), NKCC2 (160 kDa), and NaKATPase (112 kDa) of six-month-old male and female offspring. Relative abundance of renal cortical NHE3, NCC, NKCC2, NaKATPase as quantified in: male (**B**); and female (**C**) offspring. * *p* < 0.05 vs. ND/ND.

Additionally, we analyzed the levels of sodium transporters and found that renal levels of NHE3, NCC, NKCC2, and NaKATPase were not different among the four groups in males (Figure 6). However, maternal and post-weaning HF similarly increased renal NHE3, NCC, and NKCC2 protein levels in females.

### 3.6. Clock and Clock-Controlled Genes

**Figure 7:**
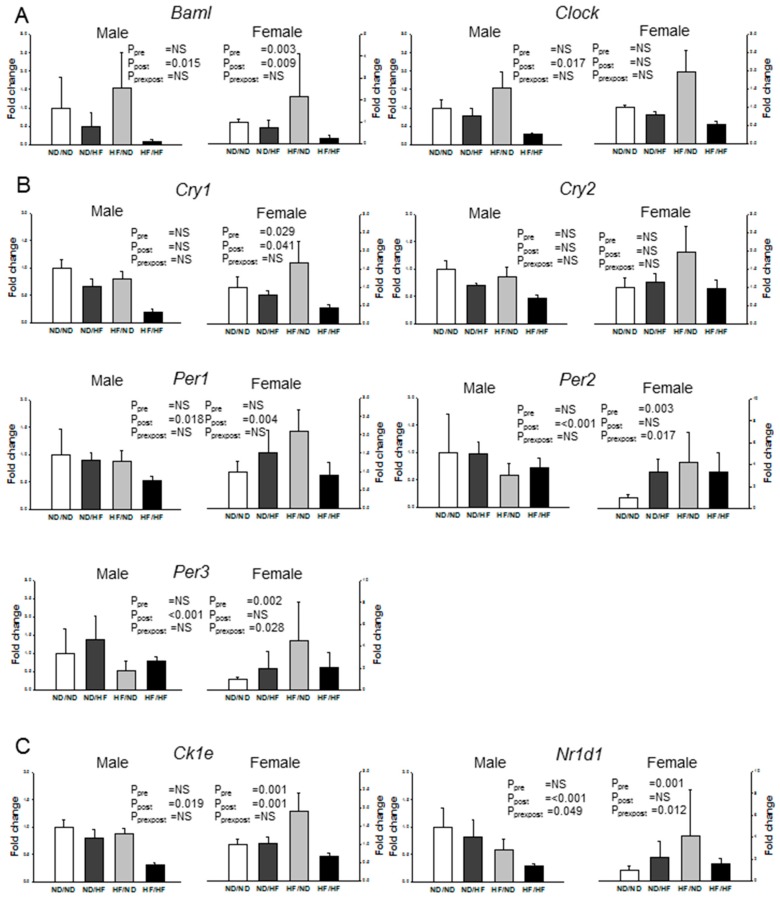
Effects of maternal and postnatal high-fat diet (HF) on mRNA levels of clock genes in male and female offspring kidneys. Relative fold changes of: (**A**) positive element *Baml* and *Clock*; (**B**) negative elements *Cry1, Per2*, and *Per3*; and (**C**) clock-controlled gene *Ck1e* and *Nr1d1* as quantified. Pre × Post, interaction of pre × post; NS, not significant; *N* (pups/L) = 5-7/3 per group.

Figure 7 represents clock and clock-controlled gene expression in offspring kidney. Maternal HF diet significantly upregulated mRNA expression of the positive element *Baml*, negative elements (*Cry1* and *Per2*), and clock-controlled gene (*Ck1e* and *Nr1d1*) in females. In males, clock and clock-controlled genes tended to be unaltered in response to maternal HF consumption. Post-weaning HF diet significantly downregulated mRNA level of most clock and clock-controlled genes in the males (All *P*_post_ < 0.05), with the exception of *Cry1* and *Cry2*. In females, post-weaning HF diet led to the downregulation of the *Baml, Ck1e, Cry1*, and *Per1*.

## 4. Discussion

This study provides insight into several sex-specific mechanisms by which maternal and post-weaning HF intake causes different renal and metabolic outcomes in adult offspring. The key findings are the following: (1) post-weaning HF diet increased body weight only in male offspring; (2) post-weaning HF diet increased systolic blood pressure in both sexes; (3) males were more vulnerable to kidney damage compared to females in response to maternal and post-weaning HF intake; (4) maternal HF altered renal transcriptome in a sex specific fashion as demonstrated by 21 and 251 DEGs in male and female offspring, respectively; (5) maternal and post-weaning HF diet-induced hypertension and renal injury relevant to oxidative stress, RAS, and sodium transporters; and (6) maternal and post-weaning HF differentially regulated renal clock-controlled genes in a sex specific manner.

We observed that maternal HF caused IUGR offspring continued to have lower body weight until one month of age in both sexes. Previous reports demonstrated that IUGR offspring, particularly those with rapid catch-up growth, have a higher risk of adult obesity and metabolic syndrome [32,33]. Although HF diets are often used to promote obesity in rodents, some authors did not find statistically differences in body weight [5]. In the present study, maternal HF elicited little effect on metabolic syndrome-like conditions (e.g., obesity and lipids) on the HF/ND offspring. However, post-weaning HF has a differential impact on the development of obesity and liver steatosis in both sexes. In males, ND/HF and HF/HF group became obese over time, with significantly elevated plasma ASL and ALT levels at six months of age. Nevertheless, post-weaning HF increased ASL and ALT levels but not body weights in female offspring. In lines with an earlier review showing that sex differences exist in obesity-related disorders [19], our results indicate that male offspring are predisposed to obesity and liver steatosis in response to HF consumption. Additionally, we observed that females exposed to HF intake tend to elicit an increased glucose AUC in IPGTT, which support the idea that impaired glucose tolerance is more prevalent in women [19]. 

Although HF diets are associated with hypertension [5], the observations of maternal HF-induced hypertension in offspring are varied [6]. Maternal HF induced responses of BP include an increase [34,35], decrease [36], or no change [34], mainly depending on strain, sex, age, measuring method, and different fatty acids compositions. In this work, we did not observe an impact from maternal HF on the development of hypertension in each sex. However, we noted that post-weaning HF similarly increased BP in either ND/HF or HF/HF group of both sexes. Previously, we and others showed pre- and post-natal insults could be independently or synergistically contributing to renal programming and programmed hypertension [8,10,20,28]. Our current study demonstrated that maternal HF did not either intensify or lessen post-weaning HF induced programmed hypertension in both sexes.

Renal injury has been reported in offspring exposed to maternal or post-weaning HF diets [24,25,32]. Consistent with previous reports showing fibrotic and epithelial-to-mesenchymal transition (EMT) markers were augmented by HF intake [30,31,37], we found that maternal and post-weaning HF increased mRNA expression of collagen I and α-SMA in the offspring kidneys of both sexes. Noteworthy, male offspring exposed to maternal plus post-weaning HF showed greater degrees of glomerular and tubulointerstitial injury and worse renal function compared to females. 

There is emerging evidence that sex differences exist in the fetal programming of kidney disease [11,38], showing that males are more vulnerable than females. The important sex-dependent differences in the developmental programming of diseases seem to be related to sex hormones [38]. Previous studies furthermore indicated that estrogen helped to protect against kidney disease while testosterone shown to be harmful to kidney health [38,39]. Whether sex hormones influence the vulnerability to protect female offspring against HF-induced programmed kidney disease deserves further clarification. Our findings in conjunction with others indicate that male offspring tend to be more vulnerable to HF-induced renal injury than females [30,31].

In line with previous studies [24,40,41], our NGS data illustrated that maternal nutrition has great effects on renal transcriptome in the developing kidney. We observed that maternal HF intake induces significant changes in renal transcriptome with female offspring being more HF-sensitive. Although sex differences have been observed in developmental programming of obesity and kidney disease [17,19], our study is the first to show sex differences of maternal HF-induced changes with a focus on renal transcriptome. Our findings are consistent with previous studies showing that more genes in the placenta were affected in females than in males in different models of nutritional programming [42,43]. Since we found that female offspring are more resilient to HF-induced obesity and kidney disease, it is possible that the increased female sensitivity to maternal HF diet might buffer the deleterious effects, resulting in a better adaptation and less impact of programming in adulthood. Our NGS data demonstrated ~20 genes in five KEGG pathways were significantly regulated in female in response to maternal HF consumption. Except defect in *Slc6a19* (encodes an amino acid transporter B°AT1) has been linked to hypertension [44], most genes are not relevant to hypertension and kidney disease. Additional studies are needed to determine whether these genes are common genes in the development of hypertension and kidney disease in other programming models. 

Emerging evidence demonstrated that an early shift in the NO-ROS balance toward reduced NO bioavailability links to programmed hypertension and kidney disease in later life [8,10,11,45]. Oxidative stress has been demonstrated as a key mediator in the pathogenesis of obesity and related disorders [5,40]. In this work, several lines of evidence implicated the role of ADMA-NO pathway related oxidative stress on programmed hypertension and kidney disease induced by HF intake. First, post-weaning HF reduced plasma level of l-arginine, a substrate for nitric oxide synthase, level in both sexes. Second, there was a significant effect of maternal and post-weaning HF with increased plasma SDMA (an indirect inhibitor of nitric oxide synthase) level and decreased l-arginine-to-ADMA ratio (a marker representing NO bioavailability) in males. Third, post-waning HF decreased plasma NOx level in male offspring. Fourth, our NGS data identified the oxidative phosphorylation is a significantly regulated KEGG pathway. As known, defective oxidative phosphorylation-induced oxidative stress play a key role in many obesity-related disorders [46]. Last, maternal and post-weaning HF increased the degrees of oxidative stress damage represented as 8-OHdG IHC staining in male offspring, which is associated with a worse renal outcome. Thus, our results demonstrated that maternal and post-weaning HF diets-induced hypertension and kidney injury along with the ROS-NO imbalance. Since sex-specific NO availability might be involved in the development of hypertension [38], and since we noted post-weaning HF induced sex-specific changes in NO availability but not BP in each sex, our findings suggested that hypertension in response to post-weaning HF intake might be independent of NO pathway in males. 

Next, we observed that HF consumption induced sex-specific alterations of the RAS and sodium transporters. However, the renoprotective mechanisms of female refractory to HF-induced kidney injury might not be related to the RAS and sodium transporters. Despite our previous study suggest sex-dependent renal programming within the RAS underling the programmed hypertension in a rat model of prenatal dexamethasone exposure [21], our present study showed that there was no sex difference on the most components of RAS in response to HF exposure. Additionally, maternal and post-weaning HF increased several sodium transporters in the female kidney, including NHE3, NCC, and NKCC2. Given that most sodium transporters are clock-controlled genes [47], and that increased expression of sodium transporters triggers programmed hypertension in various models [8,48], our observations suggest HF-induced disturbed circadian clock may induce sodium transporters to trigger sodium retention, contributing to the development of hypertension in females. However, whether HF-induced programming of kidney disease in males attributed to dysregulated RAS and sodium transporters deserve further elucidation. 

In agreement with previous studies showing that HF diets alter circadian clock function [13,49], we observed that maternal HF diet upregulated mRNA expression of the positive element (*Baml*) and negative elements (*Cry1* and *Per2*) in females. In contrast, post-weaning HF diet led to the downregulation of the *Baml, Ck1e, Cry1*, and *Per1* in female offspring kidneys. Emerging evidence suggested that clock genes such as *Baml, Ck1e, Cry1, Per1* and *Per2* play an integral role in the development of hypertension and kidney disease [50,51]. Our data would support the concept that disturbed circadian clock in the kidney, induced by maternal or post-weaning HF, may contribute to the substantial renal injury and elevation of BP. A previous report showed that the kidney is less sensitive to feeding cues compared with other tissues [52]. Our data demonstrated that the effects of HF intake on renal clock genes have a distinct sex-specific bias, with female offspring being more HF-sensitive. 

Our study has a few limitations. First, we did not examine other organs involved in obesity related diseases. The differential effects of maternal and post-weaning HF on male and female offspring may be derived from other tissues, such as the liver, fatty tissues, and vasculature. Another limitation is that clock genes expression was measured only at one point, it is not possible to infer whether the differences among the experimental groups are due to differences in gene expression degree or to a phase shift. Since HF-induced renal injury reported was related to renal accumulation of lipid in adult rats [53], additional studies are needed to elucidate whether this mechanism plays a crucial role in programmed kidney disease. Furthermore, we did not examine alterations of renal transcriptome in different windows of exposure to HF. Given that the interactions between genes and diet vary during different developmental windows, whether HF consumption leads to differentially regulated genes between diverse windows of exposure is worthy of further study. Finally, it should be noted that different nutritional insults might not use the same pathway to induce hypertension and kidney injury. Therefore, further studies should be performed using other models to determine whether the oxidative stress, RAS, sodium transporters, and circadian clock are common targets for preventing hypertension and kidney disease.

## 5. Conclusions

Thus, we conclude that maternal and post-weaning HF diet have sex-specific influences on the development of obesity, kidney injury, and hypertension. Maternal HF diet induces significant alterations in renal transcriptome with female offspring being more sensitive. Our data suggested an association among oxidative stress, RAS, sodium transporters, and circadian clock, which involved in the HF-induced hypertension and kidney injury in adult offspring. Most importantly, the coupling of maternal and post-weaning HF consumption aggravates obesity and kidney damage in males, which is associated with sex-specific renal programming. With better understanding of the sex-specific gene–diet interactions that underlie maternal and post-weaning HF-induced renal programming, our results can aid in developing effective personalized reprogramming strategies to prevent obesity and related disorders.

## Figures and Tables

**Table 1:** Quantitative real-time polymerase chain reaction primers sequences.

Gene	Forward	Reverse
*Collagen I*	5 aggcataaagggtcatcgtg 3	5 accgttgagtccatctttgc 3
α-SMA	5 gaccctgaagtatccgatagaaca 3	5 cacgcgaagctcgttatagaag 3
*Ren*	5 aacattaccagggcaactttcact 3	5 acccccttcatggtgatctg 3
*Atp6ap2*	5 gaggcagtgaccctcaacat 3	5 ccctcctcacacaacaaggt 3
*Agt*	5 gcccaggtcgcgatgat 3	5 tgtacaagatgctgagtgaggcaa 3
*Ace*	5 caccggcaaggtctgctt 3	5 cttggcatagtttcgtgaggaa 3
*Ace2*	5 acccttcttacatcagccctactg 3	5 tgtccaaaacctaccccacatat 3
*Agtr1a*	5 gctgggcaacgagtttgtct 3	5 cagtccttcagctggatcttca 3
*Agtr1b*	5 caatctggctgtggctgactt 3	5 tgcacatcacaggtccaaaga 3
*Mas1*	5 catctctcctctcggctttgtg 3	5 cctcatccggaagcaaagg 3
*Clock*	5 ccactgtacaatacgatggtgatctc 3	5 tgcggcatactggatggaat3
*Bmal1*	5 attccagggggaaccaga 3	5 gaaggtgatgaccctcttatcct 3
*Per1*	5 gcttgtgtggactgtggtagca 3	5 gccccaatccatccagttgt 3
*Per2*	5 catctgccacctcagactca 3	5 ctggtgtgacttgtatcactgct 3
*Per3*	5 tggccacagcatcagtaca 3	5 tacactgctggcactgcttc 3
*Cry1*	5 atcgtgcgcatttcacatac 3	5 tccgccattgagttctatgat 3
*Cry2*	5 gggagcatcagcaacacag 3	5 gcttccagcttgcgtttg 3
*Ck1e*	5 gcctctatcaacacccacct 3	5 ggagcccaggttgaagtaca 3
*Nr1d1*	5 ctactggctccctcacccagga 3	5 gacactcggctgctgtcttcca 3
*Rn18s*	5 gccgcggtaattccagctcca 3	5 cccgcccgctcccaagatc 3

α-SMA = α-smooth muscle actin, Ren = Renin, Atp6ap2 = Prorenin receptor (PRR), Agt = Angiotensinogen (AGT), Ace = Angiotensin converting enzyme (ACE), Ace2 = Angiotensin converting enzyme-2 (ACE2), Agtr1a = Angiotensin II type 1 receptor (AT1R), Agtr2 = Angiotensin II type 2 receptor (AT2R), Clock = Circadian locomotor output cycles kaput, Bmal1 = Brain and muscle aryl-hydrocarbon receptor nuclear translocator-like 1, Per1 = Period 1, Per2 = Period 2, Per3 =Period 3, Cry1 = Cryptochrome 1, Cry2 = Cryptochrome 2, Ck1e = Casein kinase 1 epsilon, Nr1d1 = Nuclear receptor subfamily 1, group D member 1 (also known as Rev-Erb-alpha), Rn18s = 18S ribosomal RNA (r18S).

**Table 2:** Weights and metabolic parameters in offspring at six months of age.

Groups		ND/ND	ND/HF	HF/ND	HF/HF	*p* Value		
Number		M = 6; F = 6	M = 6; F = 6	M = 6; F = 6	M = 7; F = 6	Pre	Post	Pre × Post
Body weight (g)	Male	641 ± 39	785 ± 39 *	677 ± 21	813 ± 41*^,$^	NS	0.001	NS
	Female	372 ± 17	362 ± 19	355 ± 14	372 ± 10	NS	NS	NS
Left kidney (LK) weight (g)	Male	2.16 ± 0.14	2.12 ± 0.08	2.26 ± 0.08	2.08 ± 0.07	NS	NS	NS
	Female	1.28 ± 0.04	1.45 ± 0.1	1.37 ± 0.05	1.38 ± 0.01	NS	NS	NS
LK weight/100 g BW	Male	0.34 ± 0.02	0.27 ± 0.01 *	0.33 ± 0.01 ^#^	0.26 ± 0.01*^,$^	NS	<0.001	NS
	Female	0.35 ± 0.02	0.4 ± 0.02	0.39 ± 0.01	0.37 ± 0.01	NS	NS	0.021
AST (U/L)	Male	88 ± 11	308 ± 58 *	82 ± 10 ^#^	145 ± 19 ^#^	0.019	<0.001	0.028
	Female	73 ± 3	160 ± 24	83 ± 12	82 ± 8	0.026	0.007	0.006
ALT (U/L)	Male	27 ± 3	196 ± 52 *	23 ± 2 ^#^	66 ± 17 ^#^	0.031	0.002	0.041
	Female	19 ± 2	67 ± 13	22 ± 3	30 ± 4	0.026	0.001	0.009
Total cholesterol (mg/dL)	Male	71 ± 8	82 ± 7	58 ± 5	65 ± 5	0.027	NS	NS
	Female	81 ± 9	95 ± 4	104 ± 7	95 ± 16	NS	NS	NS
Triglyceride (mg/dL)	Male	101 ± 26	61 ± 13	105 ± 9	87 ± 12	NS	NS	NS
	Female	97 ± 23	58 ± 9	120 ± 23 ^#^	60 ± 8	NS	NS	NS
HDL (mg/dL)	Male	43 ± 4	49 ± 6	34 ± 4	42 ± 4	NS	NS	NS
	Female	39 ± 4	52 ± 2	59 ± 4	58 ± 10	NS	NS	NS
Glucose (mg/dL)	Male	81 ± 2	91 ± 3	93 ± 4	81 ± 3	NS	NS	NS
	Female	75 ± 4	76 ± 1	73 ± 2	62 ± 3 ^#^	NS	NS	NS
IPGTT (AUC, mg/dL·120 min)	Male	22,071 ± 1354	23,923 ± 2345	23,498 ± 2286	25,922 ± 1973	-	-	-
	Female	26,420 ± 1406	31,389 ± 1773 *	26,890 ± 1820	26,949 ± 2416	-	-	-

AST, aspartate transaminase; ALT, alanine aminotransferase; HDL, high-density lipoprotein; IPGTT, intraperitoneal glucose tolerance test; AUC, area under curve; ND, normal diet; HF, high-fat diet; NS, not significant; -, not done; * *P* < 0.05 vs. ND/ND; ^#^ *P* < 0.05 vs. ND/HF; ^$^ *P* < 0.05 vs. HF/ND; *N* (pups/L) = 5-7/3 per group.

**Table 3:** Significantly regulated Kyoto Encyclopedia of Genes and Genomes (KEGG) pathways in the one-week-old offspring kidneys exposed to maternal high-fat (HF) consumption.

KEGG Pathway	Count	Gene Symbol	*p*-Value	Benjamini
Male				
Protein digestion and absorption	2	*Slc15a1, Slc6a19*	5.6 × 10^−2^	5.6 × 10^−2^
Female				
Oxidative phosphorylation	5	*Atp5j2, Atp6v0d2, Ndufa5, Cox6c, Cox7c*	1.6 × 10^−2^	1.6 × 10^−2^
Protein digestion and absorption	4	*Slc15a1, Slc6a19, Slc7a7, Slc7a8*	2.2 × 10^−2^	2.2 × 10^−2^
Metabolic pathways	16	*Dhcr24, Abat, Atp5j2, Atp6v0d2, C1qalt1c1, Mgat4c, Ndufa5, Alox15, Cyp24a1, Cox6c, Cox7c, Dse, Dqkq, Gatm, Hykk, Polr2k*	2.3 × 10^−2^	2.3 × 10^−2^
Ribosome	5	*Mrpl33, Mrps18c, Rpl22l1, Rpl30, LOC100362027*	2.9 × 10^−2^	2.9 × 10^−2^
Cardiac muscle contraction	3	*Cacna2d2, Cox6c, Cox7c*	9.9 × 10^−2^	9.9 × 10^−2^

The top results, sorted by enrichment probability value and the Benjamini–Hochberg multiple testing correction for each Kyoto Encyclopedia of Genes and Genomes (KEGG) pathway, are reported.

**Table 4:** Plasma levels of l-arginine, l-citrulline, ADMA, SDMA, and NO in offspring at six months of age.

Groups		ND/ND	ND/HF	HF/ND	HF/HF	P Value		
Number		M = 5; F = 6	M = 6; F = 6	M = 6; F = 6	M = 7; F = 6	Pre	Post	Pre × Post
l-Citrulline (μM)	Male	42.4 ± 2.2	42.0 ± 2.0	41.6 ± 1.6	46.2 ± 1.9	NS	NS	NS
	Female	48.7 ± 4.1	61.7 ± 9.5	44.5 ± 4.8	62.9 ± 4.7	NS	0.019	NS
l-Arginine (μM)	Male	168.0 ± 15.9	46.1 ± 12.8 *	172.0 ± 9.5 ^#^	101.8 ± 15.5	0.041	<0.001	NS
	Female	152.4 ± 27.7	120.8 ± 23.4	179.0 ± 10.2	119.6 ± 5.2	NS	0.027	NS
ADMA (μM)	Male	1.02 ± 0.03	0.92 ± 0.03	1.23 ± 0.03 ^#^	1.03 ± 0.07	0.001	0.003	NS
	Female	1.45 ± 0.09	1.25 ± 0.07	1.38 ± 0.03	1.1 ± 0.04 *^,$^	NS	0.001	NS
SDMA(μM)	Male	0.43 ± 0.03	0.55 ± 0.02	0.67 ± 0.03 *	0.53 ± 0.02	0.001	NS	<0.001
	Female	0.72 ± 0.08	0.65 ± 0.03	0.68 ± 0.05	0.5 ± 0.03*	NS	0.028	NS
l-Arginine-to-ADMA ratio (μM/μM)	Male	165.7 ± 15.1	49.7 ± 13.9 *	138.6 ± 7.7 ^#^	101.7 ± 17.6*	NS	<0.001	0.011
	Female	107.2 ± 20.8	93.3 ± 16.0	129.1 ± 9.6	107.9 ± 3.0	NS	NS	NS
NOx (NO2^−^ + NO^−^) (μM)	Male	218.6 ± 15.1	167.5 ± 5.1	195.7 ± 6.6	178 ± 7.4	NS	0.001	NS
	Female	172.8 ± 16.5	161.5 ± 17.6	176.6 ± 16.5	159.1 ± 24.5	NS	NS	NS

ADMA, asymmetric dimethylarginine; SDMA, symmetric dimethylarginine; ND, normal diet; HF, high-fat diet; Pre × Post, interaction of pre × post; NS, not significant; * *P* < 0.05 vs. ND/ND; ^#^ *P* < 0.05 vs. ND/HF; ^$^ *P* < 0.05 vs. HF/ND; *N* (pups/L) = 5-7/3 per group.

## Supplementary Materials

The following are available online at www.mdpi.com/2072-6643/9/4/357/s1, Table S1: List of the 21 differentially expressed genes that are induced by maternal high-fat (HF) exposure in 1-week-old male offspring kidney, Table S2: List of the 251 differentially expressed genes that are induced by maternal high-fat (HF) exposure in 1-week-old female offspring kidney, Table S3: List of the 91 differentially expressed genes in the kidney of control male offspring vs. control female offspring at 1 week of age, Table S4: List of the 9 differentially expressed genes in the kidney of maternal high-fat (HF)-treated male offspring vs. maternal HF-treated female offspring at 1 week of age.
