# Supplementary material for: High Fat Diets Sex-Specifically Affect the Renal Transcriptome and Program Obesity, Kidney Injury, and Hypertension in the Offspring
Source: Nutrients. 2017 Apr 3;9(4):357. doi: 10.3390/nu9040357 (PMC5409696; doi:10.3390/nu9040357)
Supplement: Supplementary file 1 [file nutrients-09-00357-s001.zip › Supplementary materials.doc]

Table S1. List of the 21 differentially expressed genes that are induced by maternal high-fat (HF) exposure in 1-week-old male offspring kidney.

| Gene_ID | Gene Symbol | Fold changes |
| --- | --- | --- |
| ENSRNOG00000039386 | LOC100365571 | 9198 |
| ENSRNOG00000010714 | Fgl1 | 4016 |
| ENSRNOG00000002911 | Alb | 22.48 |
| ENSRNOG00000023320 | Tspan1 | 4.06 |
| ENSRNOG00000033932 | - | 3.19 |
| ENSRNOG00000002889 | Afp | 0.336 |
| ENSRNOG00000013410 | Spink1 | 0.284 |
| ENSRNOG00000025601 | D3ZUC6_RAT | 0.260 |
| ENSRNOG00000026501 | Slc6a19 | 0.251 |
| ENSRNOG00000001656 | Kcnj15 | 0.221 |
| ENSRNOG00000030487 | Lrp2 | 0.207 |
| ENSRNOG00000021809 | Gpx3 | 0.197 |
| ENSRNOG00000001796 | Dgkg | 0.185 |
| ENSRNOG00000003134 | Slc4a4 | 0.178 |
| ENSRNOG00000011598 | Slc15a1 | 0.177 |
| ENSRNOG00000029047 | Cubn | 0.175 |
| ENSRNOG00000008312 | Stra6 | 0.160 |
| ENSRNOG00000014587 | Mid2 | 0.112 |
| ENSRNOG00000028008 | Slc5a12 | 0.099 |
| ENSRNOG00000028765 | LOC100365656 | 1.8E-04 |
| ENSRNOG00000011040 | - | 9.9E-05 |

Genes are sorted by fold changes in descending order.

Table S2. List of the 251 differentially expressed genes that are induced by maternal high-fat (HF) exposure in 1-week-old female offspring kidney.

| Gene_ID | Gene Symbol | Fold changes |
| --- | --- | --- |
| ENSRNOG00000034422 | *SNORD81* | 3624930 |
| ENSRNOG00000035760 | *SNORD47* | 3570520 |
| ENSRNOG00000035425 | *U6* | 1010970 |
| ENSRNOG00000032918 | *F1LZ76_RAT* | 182017 |
| ENSRNOG00000037032 | *F1M890_RAT* | 21930 |
| ENSRNOG00000030214 | *D3ZDE9_RAT* | 16339 |
| ENSRNOG00000023895 | *F1M158_RAT* | 7162 |
| ENSRNOG00000004149 | *Mgat4c* | 5445 |
| ENSRNOG00000022052 | *RGD1562667* | 4914 |
| ENSRNOG00000021896 | *Mmp20* | 4709 |
| ENSRNOG00000029957 | *Zfp51* | 13.54 |
| ENSRNOG00000039096 | *F1M0C9_RAT* | 9.59 |
| ENSRNOG00000029433 | *F1LZ97_RAT* | 8.51 |
| ENSRNOG00000032943 | *F1LTA3_RAT* | 8.37 |
| ENSRNOG00000017986 | *Zfp458* | 8.32 |
| ENSRNOG00000032158 | *F1M6H3_RAT* | 8.17 |
| ENSRNOG00000007372 | *Casp1* | 7.79 |
| ENSRNOG00000027331 | *PPE1_RAT* | 7.76 |
| ENSRNOG00000022218 | *D3ZAF8_RAT* | 7.65 |
| ENSRNOG00000029602 | *F1LX69_RAT* | 7.55 |
| ENSRNOG00000033551 | *D3ZDY7_RAT* | 7.23 |
| ENSRNOG00000042285 | *F1LZ32_RAT* | 7.07 |
| ENSRNOG00000017945 | *Serf1* | 6.64 |
| ENSRNOG00000013464 | *Spink3* | 6.59 |
| ENSRNOG00000028451 | *Cox7b* | 6.48 |
| ENSRNOG00000020296 | *Usmg5* | 6.39 |
| ENSRNOG00000009782 | *Efcab7* | 6.28 |
| ENSRNOG00000002536 | *C1galt1c1* | 6.23 |
| ENSRNOG00000013410 | *Spink1* | 6.17 |
| ENSRNOG00000011817 | *Rpl22l1* | 6.11 |
| ENSRNOG00000025768 | *Clk1* | 6.00 |
| ENSRNOG00000002835 | *Luc7l3* | 5.91 |
| ENSRNOG00000005858 | *Kap* | 5.83 |
| ENSRNOG00000015410 | *Aspn* | 5.80 |
| ENSRNOG00000026569 | *LOC100364115* | 5.79 |
| ENSRNOG00000010408 | *LOC100361574* | 5.65 |
| ENSRNOG00000031291 | *D3ZF90_RAT* | 5.63 |
| ENSRNOG00000016330 | *Rbm12b* | 5.45 |
| ENSRNOG00000001829 | *Ube2v2* | 5.44 |
| ENSRNOG00000022252 | *Kbtbd3* | 5.40 |
| ENSRNOG00000004419 | *D3ZVF4_RAT* | 5.36 |
| ENSRNOG00000004754 | *D4A866_RAT* | 5.35 |
| ENSRNOG00000042826 | *Zfp52* | 5.22 |
| ENSRNOG00000009116 | *Itgb3bp* | 5.20 |
| ENSRNOG00000010189 | *Rps24* | 5.18 |
| ENSRNOG00000010461 | *Gpx8* | 5.14 |
| ENSRNOG00000028582 | *F1M163_RAT* | 5.12 |
| ENSRNOG00000029792 | *Ogn* | 5.11 |
| ENSRNOG00000026643 | *Chordc1* | 5.05 |
| ENSRNOG00000005975 | *LOC100364549* | 5.04 |
| ENSRNOG00000032925 | *LOC100360432* | 5.03 |
| ENSRNOG00000017318 | *Zfp322a* | 5.00 |
| ENSRNOG00000009841 | *E9PTD6_RAT* | 4.98 |
| ENSRNOG00000007124 | *Krcc1* | 4.86 |
| ENSRNOG00000008309 | *Rpa3* | 4.84 |
| ENSRNOG00000030894 | *Slco1a6* | 4.83 |
| ENSRNOG00000009462 | *Ccdc90b* | 4.82 |
| ENSRNOG00000030237 | *Cox7c* | 4.81 |
| ENSRNOG00000010921 | *Taf1d* | 4.81 |
| ENSRNOG00000043289 | *LOC100125368* | 4.76 |
| ENSRNOG00000023862 | *-* | 4.74 |
| ENSRNOG00000018208 | *D3ZCM1_RAT* | 4.73 |
| ENSRNOG00000015711 | *Cetn3* | 4.73 |
| ENSRNOG00000012512 | *NEXN_RAT* | 4.71 |
| ENSRNOG00000014618 | *Med31* | 4.67 |
| ENSRNOG00000015332 | *Thoc1* | 4.66 |
| ENSRNOG00000006121 | *D3ZIB7_RAT* | 4.64 |
| ENSRNOG00000000891 | *F1LYH2_RAT* | 4.64 |
| ENSRNOG00000010807 | *Cox6c* | 4.63 |
| ENSRNOG00000015516 | *Cbwd1* | 4.56 |
| ENSRNOG00000028328 | *Tyw3* | 4.55 |
| ENSRNOG00000011675 | *Zfp40* | 4.46 |
| ENSRNOG00000005123 | *Ttc35* | 4.41 |
| ENSRNOG00000001317 | *Zfp68* | 4.40 |
| ENSRNOG00000010640 | *Agtr1b* | 4.37 |
| ENSRNOG00000013803 | *Hells* | 4.35 |
| ENSRNOG00000011008 | *Bet1* | 4.35 |
| ENSRNOG00000015523 | *RGD1308147* | 4.31 |
| ENSRNOG00000042126 | *Lsm5* | 4.25 |
| ENSRNOG00000032258 | *RGD1308695* | 4.22 |
| ENSRNOG00000002280 | *Sh3bgrl* | 4.17 |
| ENSRNOG00000004268 | *Zfp386* | 4.16 |
| ENSRNOG00000028585 | *Tceal8* | 4.08 |
| ENSRNOG00000010891 | *Lrrcc1* | 4.08 |
| ENSRNOG00000014501 | *Zfml* | 4.07 |
| ENSRNOG00000008504 | *Lsm8* | 4.07 |
| ENSRNOG00000005698 | *Ndufa5* | 4.03 |
| ENSRNOG00000004737 | *Cd48* | 4.03 |
| ENSRNOG00000009297 | *Chchd1* | 4.02 |
| ENSRNOG00000029191 | *LOC685067* | 4.01 |
| ENSRNOG00000004829 | *Immp1l* | 4.00 |
| ENSRNOG00000039551 | *Rdh14* | 3.95 |
| ENSRNOG00000006244 | *Lztfl1* | 3.95 |
| ENSRNOG00000017469 | *Anxa1* | 3.93 |
| ENSRNOG00000027049 | *Atp5j2* | 3.88 |
| ENSRNOG00000002178 | *Mrps18c* | 3.87 |
| ENSRNOG00000000728 | *Clic2* | 3.85 |
| ENSRNOG00000017310 | *Rnpc3* | 3.85 |
| ENSRNOG00000038960 | *RGD1309362* | 3.81 |
| ENSRNOG00000008744 | *Cops2* | 3.81 |
| ENSRNOG00000000120 | *Tceb1* | 3.81 |
| ENSRNOG00000017784 | *RGD1306520* | 3.75 |
| ENSRNOG00000009156 | *Tra2a* | 3.75 |
| ENSRNOG00000030520 | *Sarnp* | 3.72 |
| ENSRNOG00000003865 | *Tmigd1* | 3.72 |
| ENSRNOG00000027690 | *Eif3e* | 3.70 |
| ENSRNOG00000025388 | *LOC100363539* | 3.67 |
| ENSRNOG00000032274 | *-* | 3.65 |
| ENSRNOG00000010388 | *Slc21a4* | 3.64 |
| ENSRNOG00000013756 | *Snrnp48* | 3.63 |
| ENSRNOG00000020315 | *Taf13* | 3.62 |
| ENSRNOG00000031353 | *Ust5r* | 3.61 |
| ENSRNOG00000014380 | *D4ACK9_RAT* | 3.60 |
| ENSRNOG00000006926 | *Atp6v0d2* | 3.57 |
| ENSRNOG00000024372 | *Cwf19l2* | 3.57 |
| ENSRNOG00000004282 | *Arl4a* | 3.55 |
| ENSRNOG00000013002 | *Gpbp1* | 3.54 |
| ENSRNOG00000018153 | *Rbm5* | 3.54 |
| ENSRNOG00000036971 | *RGD1566320* | 3.52 |
| ENSRNOG00000007875 | *Actr6* | 3.51 |
| ENSRNOG00000043394 | *LOC100365201* | 3.50 |
| ENSRNOG00000014582 | *Mrpl18* | 3.46 |
| ENSRNOG00000010147 | *Pdcd10* | 3.45 |
| ENSRNOG00000008891 | *Ift20* | 3.44 |
| ENSRNOG00000023226 | *S100a10* | 3.42 |
| ENSRNOG00000020204 | *Srp19* | 3.40 |
| ENSRNOG00000012738 | *Eif3m* | 3.38 |
| ENSRNOG00000011558 | *Sub1* | 3.38 |
| ENSRNOG00000031421 | *Eif1a* | 3.37 |
| ENSRNOG00000000824 | *Dse* | 3.36 |
| ENSRNOG00000003242 | *Gulp1* | 3.36 |
| ENSRNOG00000012778 | *Magoh* | 3.35 |
| ENSRNOG00000023651 | *F1LTQ4_RAT* | 3.33 |
| ENSRNOG00000022910 | *Emcn* | 3.32 |
| ENSRNOG00000032825 | *LOC100365668* | 3.30 |
| ENSRNOG00000007512 | *Srp14* | 3.27 |
| ENSRNOG00000000396 | *Ddx50* | 3.23 |
| ENSRNOG00000019107 | *Q7TPL2_RAT* | 3.19 |
| ENSRNOG00000010453 | *Klhl7* | 3.19 |
| ENSRNOG00000007203 | *Psmc6* | 3.18 |
| ENSRNOG00000009888 | *Timm8b* | 3.16 |
| ENSRNOG00000009451 | *Prpf38a* | 3.16 |
| ENSRNOG00000012100 | *Ssbp1* | 3.14 |
| ENSRNOG00000016133 | *Sumo1* | 3.11 |
| ENSRNOG00000014624 | *Selk* | 3.11 |
| ENSRNOG00000029262 | *D3Z9G9_RAT* | 3.09 |
| ENSRNOG00000006411 | *Cav2* | 3.04 |
| ENSRNOG00000002662 | *RGD1562502* | 3.01 |
| ENSRNOG00000026660 | *D4A8W1_RAT* | 3.00 |
| ENSRNOG00000002947 | *Dpt* | 3.00 |
| ENSRNOG00000003901 | *Ccdc104* | 2.99 |
| ENSRNOG00000007336 | *Churc1* | 2.98 |
| ENSRNOG00000017447 | *Eif2s2* | 2.95 |
| ENSRNOG00000021206 | *Pla2g16* | 2.95 |
| ENSRNOG00000017059 | *Cetn2* | 2.89 |
| ENSRNOG00000013178 | *Cmip* | 0.339 |
| ENSRNOG00000008981 | *Pdcd6ip* | 0.334 |
| ENSRNOG00000000568 | *Slc29a3* | 0.334 |
| ENSRNOG00000002864 | *Nacc1* | 0.334 |
| ENSRNOG00000004964 | *Erbb3* | 0.332 |
| ENSRNOG00000014530 | *Nav2* | 0.331 |
| ENSRNOG00000008345 | *Clcn6* | 0.330 |
| ENSRNOG00000013954 | *Alpl* | 0.328 |
| ENSRNOG00000009957 | *Slc25a13* | 0.327 |
| ENSRNOG00000020420 | *Pklr* | 0.326 |
| ENSRNOG00000043503 | *Ehd1* | 0.325 |
| ENSRNOG00000032609 | *-* | 0.325 |
| ENSRNOG00000008895 | *Hnf4a* | 0.323 |
| ENSRNOG00000014191 | *Zfp395* | 0.318 |
| ENSRNOG00000010196 | *Glb1* | 0.318 |
| ENSRNOG00000005108 | *Wfs1* | 0.315 |
| ENSRNOG00000020440 | *Fads2* | 0.314 |
| ENSRNOG00000018835 | *Notch2* | 0.312 |
| ENSRNOG00000033389 | *Susd2* | 0.310 |
| ENSRNOG00000007467 | *Ace* | 0.310 |
| ENSRNOG00000031232 | *Nrp2* | 0.309 |
| ENSRNOG00000006718 | *Rbm33* | 0.306 |
| ENSRNOG00000010526 | *Dennd1a* | 0.303 |
| ENSRNOG00000029141 | *RGD1564942* | 0.300 |
| ENSRNOG00000018607 | *Slc12a3* | 0.295 |
| ENSRNOG00000033206 | *Entpd5* | 0.295 |
| ENSRNOG00000005130 | *Ogdh* | 0.292 |
| ENSRNOG00000030121 | *Enpep* | 0.289 |
| ENSRNOG00000008322 | *E9PTI4_RAT* | 0.281 |
| ENSRNOG00000004919 | *Gns* | 0.281 |
| ENSRNOG00000001656 | *Kcnj15* | 0.279 |
| ENSRNOG00000025498 | *Crb2* | 0.275 |
| ENSRNOG00000007927 | *Mettl7b* | 0.272 |
| ENSRNOG00000000555 | *Eif4ebp2* | 0.271 |
| ENSRNOG00000036806 | *Psme3* | 0.271 |
| ENSRNOG00000006663 | *Usp2* | 0.269 |
| ENSRNOG00000014613 | *Ddah1* | 0.267 |
| ENSRNOG00000023152 | *Tmem201* | 0.263 |
| ENSRNOG00000000488 | *Hmga1* | 0.263 |
| ENSRNOG00000023587 | *DHTK1_RAT* | 0.263 |
| ENSRNOG00000023162 | *Car14* | 0.262 |
| ENSRNOG00000019183 | *Alox15* | 0.259 |
| ENSRNOG00000008988 | *Cyp2d2* | 0.259 |
| ENSRNOG00000017762 | *Mucdhl* | 0.258 |
| ENSRNOG00000013419 | *Agphd1* | 0.255 |
| ENSRNOG00000000187 | *Csf2rb* | 0.255 |
| ENSRNOG00000033932 | *-* | 0.254 |
| ENSRNOG00000011136 | *Osr2* | 0.251 |
| ENSRNOG00000014311 | *Slc7a8* | 0.247 |
| ENSRNOG00000001796 | *Dgkg* | 0.247 |
| ENSRNOG00000034200 | *F1M585_RAT* | 0.246 |
| ENSRNOG00000010296 | *Slc7a7* | 0.245 |
| ENSRNOG00000003835 | *Slc43a2* | 0.244 |
| ENSRNOG00000000590 | *Naglt1* | 0.243 |
| ENSRNOG00000015156 | *Gal* | 0.240 |
| ENSRNOG00000006787 | *Dhcr24* | 0.240 |
| ENSRNOG00000002636 | *Abat* | 0.239 |
| ENSRNOG00000025167 | *Sema4b* | 0.237 |
| ENSRNOG00000013729 | *RGD1306271* | 0.237 |
| ENSRNOG00000000245 | *Slc16a6* | 0.234 |
| ENSRNOG00000007034 | *Hipk2* | 0.233 |
| ENSRNOG00000030355 | *LOC100125385* | 0.232 |
| ENSRNOG00000004882 | *Capn6* | 0.230 |
| ENSRNOG00000010451 | *Slc34a3* | 0.221 |
| ENSRNOG00000000168 | *Gatm* | 0.220 |
| ENSRNOG00000015763 | *Cml3* | 0.218 |
| ENSRNOG00000015260 | *Lpar3* | 0.216 |
| ENSRNOG00000017917 | *Pcdh24* | 0.206 |
| ENSRNOG00000022054 | *Paqr7* | 0.205 |
| ENSRNOG00000009448 | *D3ZD40_RAT* | 0.201 |
| ENSRNOG00000026501 | *Slc6a19* | 0.189 |
| ENSRNOG00000037824 | *Q62676_RAT* | 0.187 |
| ENSRNOG00000003134 | *Slc4a4* | 0.180 |
| ENSRNOG00000026177 | *D4A3J6_RAT* | 0.171 |
| ENSRNOG00000017438 | *Gfra1* | 0.170 |
| ENSRNOG00000002889 | *Afp* | 0.168 |
| ENSRNOG00000031333 | *-* | 0.161 |
| ENSRNOG00000028274 | *D4A352_RAT* | 0.157 |
| ENSRNOG00000030487 | *Lrp2* | 0.157 |
| ENSRNOG00000002832 | *Slc16a2* | 0.155 |
| ENSRNOG00000011598 | *Slc15a1* | 0.155 |
| ENSRNOG00000006235 | *Nell2* | 0.153 |
| ENSRNOG00000014117 | *Hmox1* | 0.148 |
| ENSRNOG00000015061 | *Igfals* | 0.143 |
| ENSRNOG00000008312 | *Stra6* | 0.120 |
| ENSRNOG00000005392 | *Ngfr* | 0.114 |
| ENSRNOG00000031207 | *LOC500035* | 0.107 |
| ENSRNOG00000029047 | *Cubn* | 0.091 |
| ENSRNOG00000006709 | *Pzp* | 0.073 |
| ENSRNOG00000008976 | *F1M014_RAT* | 0.064 |
| ENSRNOG00000013062 | *Cyp24a1* | 0.046 |
| ENSRNOG00000012821 | *Lect1* | 2.43E-04 |
| ENSRNOG00000038270 | *RGD1563231* | 1.65E-04 |
| ENSRNOG00000008394 | *Prg2* | 8.83E-05 |
| ENSRNOG00000027645 | *Tmem90a* | 4.81E-05 |
| ENSRNOG00000024511 | *D3ZM80_RAT* | 4.00E-05 |

Genes are sorted by fold changes in descending order.

Table S3. List of the 91 differentially expressed genes in the kidney of control male offspring vs. control female offspring at 1 week of age.

| Gene_ID | Gene Symbol | Fold changes |
| --- | --- | --- |
| ENSRNOG00000036024 | *SNORD29* | 434654 |
| ENSRNOG00000035760 | *SNORD47* | 252837 |
| ENSRNOG00000040389 | *-* | 242395 |
| ENSRNOG00000035336 | *SNORD77* | 176133 |
| ENSRNOG00000040987 | *SNORA68* | 68796 |
| ENSRNOG00000032918 | *F1LZ76_RAT* | 22145 |
| ENSRNOG00000039655 | *F1M9K0_RAT* | 9750 |
| ENSRNOG00000032811 | *-* | 1718 |
| ENSRNOG00000032284 | *D3ZNN2_RAT* | 1593 |
| ENSRNOG00000033725 | *RGD1564325* | 1199 |
| ENSRNOG00000036750 | *F1LWM6_RAT* | 1065 |
| ENSRNOG00000022328 | *-* | 806 |
| ENSRNOG00000022693 | *D3ZBQ0_RAT* | 794 |
| ENSRNOG00000031879 | *RGD1561928* | 488 |
| ENSRNOG00000003895 | *Rgs1* | 472 |
| ENSRNOG00000013410 | *Spink1* | 12.14 |
| ENSRNOG00000017986 | *Zfp458* | 7.62 |
| ENSRNOG00000032943 | *F1LTA3_RAT* | 7.52 |
| ENSRNOG00000016330 | *Rbm12b* | 7.28 |
| ENSRNOG00000002835 | *Luc7l3* | 6.58 |
| ENSRNOG00000033551 | *D3ZDY7_RAT* | 6.39 |
| ENSRNOG00000026569 | *LOC100364115* | 5.99 |
| ENSRNOG00000017318 | *Zfp322a* | 5.81 |
| ENSRNOG00000015410 | *Aspn* | 5.70 |
| ENSRNOG00000026643 | *Chordc1* | 5.40 |
| ENSRNOG00000009782 | *Efcab7* | 5.27 |
| ENSRNOG00000025768 | *Clk1* | 5.19 |
| ENSRNOG00000031291 | *D3ZF90_RAT* | 5.06 |
| ENSRNOG00000020296 | *Usmg5* | 4.84 |
| ENSRNOG00000032258 | *RGD1308695* | 4.79 |
| ENSRNOG00000000891 | *F1LYH2_RAT* | 4.76 |
| ENSRNOG00000019127 | *Znf606* | 4.69 |
| ENSRNOG00000011817 | *Rpl22l1* | 4.65 |
| ENSRNOG00000004754 | *D4A866_RAT* | 4.58 |
| ENSRNOG00000002536 | *C1galt1c1* | 4.55 |
| ENSRNOG00000001829 | *Ube2v2* | 4.53 |
| ENSRNOG00000038960 | *RGD1309362* | 4.50 |
| ENSRNOG00000007124 | *Krcc1* | 4.44 |
| ENSRNOG00000006244 | *Lztfl1* | 4.38 |
| ENSRNOG00000010921 | *Taf1d* | 4.34 |
| ENSRNOG00000009841 | *E9PTD6_RAT* | 4.34 |
| ENSRNOG00000001317 | *Zfp68* | 4.33 |
| ENSRNOG00000012512 | *NEXN_RAT* | 4.26 |
| ENSRNOG00000005123 | *Ttc35* | 4.25 |
| ENSRNOG00000032825 | *LOC100365668* | 4.17 |
| ENSRNOG00000008309 | *Rpa3* | 4.10 |
| ENSRNOG00000022845 | *Cep70* | 4.09 |
| ENSRNOG00000015332 | *Thoc1* | 4.07 |
| ENSRNOG00000014501 | *Zfml* | 4.07 |
| ENSRNOG00000014031 | *Esco1* | 4.06 |
| ENSRNOG00000009156 | *Tra2a* | 4.01 |
| ENSRNOG00000015711 | *Cetn3* | 3.98 |
| ENSRNOG00000029792 | *Ogn* | 3.95 |
| ENSRNOG00000010408 | *LOC100361574* | 3.95 |
| ENSRNOG00000004419 | *D3ZVF4_RAT* | 3.90 |
| ENSRNOG00000024372 | *Cwf19l2* | 3.90 |
| ENSRNOG00000011008 | *Bet1* | 3.85 |
| ENSRNOG00000009116 | *Itgb3bp* | 3.84 |
| ENSRNOG00000018153 | *Rbm5* | 3.82 |
| ENSRNOG00000008744 | *Cops2* | 3.79 |
| ENSRNOG00000003865 | *Tmigd1* | 3.60 |
| ENSRNOG00000030894 | *Slco1a6* | 3.56 |
| ENSRNOG00000020315 | *Taf13* | 3.54 |
| ENSRNOG00000002280 | *Sh3bgrl* | 3.53 |
| ENSRNOG00000008504 | *Lsm8* | 3.47 |
| ENSRNOG00000028582 | *F1M163_RAT* | 3.46 |
| ENSRNOG00000010891 | *Lrrcc1* | 3.22 |
| ENSRNOG00000017917 | *Pcdh24* | 0.279 |
| ENSRNOG00000017438 | *Gfra1* | 0.260 |
| ENSRNOG00000038622 | *LOC100359484* | 0.258 |
| ENSRNOG00000007545 | *Angptl4* | 0.252 |
| ENSRNOG00000029047 | *Cubn* | 0.251 |
| ENSRNOG00000022054 | *Paqr7* | 0.232 |
| ENSRNOG00000006709 | *Pzp* | 0.222 |
| ENSRNOG00000012067 | *Fam111a* | 0.149 |
| ENSRNOG00000005392 | *Ngfr* | 0.141 |
| ENSRNOG00000009565 | *Pdk4* | 0.132 |
| ENSRNOG00000029954 | *-* | 0.125 |
| ENSRNOG00000032609 | *-* | 0.115 |
| ENSRNOG00000031211 | *ACSM5_RAT* | 0.104 |
| ENSRNOG00000008976 | *F1M014_RAT* | 0.095 |
| ENSRNOG00000033932 | *-* | 0.086 |
| ENSRNOG00000013062 | *Cyp24a1* | 0.071 |
| ENSRNOG00000031333 | *-* | 0.060 |
| ENSRNOG00000033365 | *A4FTZ7_RAT* | 2.44E-03 |
| ENSRNOG00000000491 | *Spdef* | 2.06E-03 |
| ENSRNOG00000026462 | *D3ZN59_RAT* | 1.92E-03 |
| ENSRNOG00000022567 | *RGD1560166* | 1.90E-03 |
| ENSRNOG00000029276 | *Hist1h2ai* | 7.21E-04 |
| ENSRNOG00000041421 | *-* | 1.68E-05 |
| ENSRNOG00000035271 | *SNORD37* | 2.18E-06 |

Genes are sorted by fold changes in descending order.

Table S4. List of the 9 differentially expressed genes in the kidney of maternal high-fat (HF)-treated male offspring vs. maternal HF-treated female offspring at 1 week of age.

| Gene_ID | Gene Symbol | Fold changes |
| --- | --- | --- |
| ENSRNOG00000039202 | *F1M126_RAT* | 1102 |
| ENSRNOG00000001980 | *Ugt2b36* | 808 |
| ENSRNOG00000012067 | *Fam111a* | 0.222 |
| ENSRNOG00000021896 | *Mmp20* | 2.07E-03 |
| ENSRNOG00000025571 | *LOC681872* | 1.58E-03 |
| ENSRNOG00000000510 | *Clps* | 1.33E-03 |
| ENSRNOG00000015293 | *Tcte3* | 1.23E-03 |
| ENSRNOG00000036083 | *5_8S_rRNA* | 4.97E-05 |
| ENSRNOG00000034328 | *SNORA24* | 2.53E-05 |

Genes are sorted by fold changes in descending order.
